# 3-Hydroxypyruvate Destabilizes Hypoxia Inducible Factor and Induces Angiostasis

**DOI:** 10.1167/iovs.18-24120

**Published:** 2018-07

**Authors:** Charandeep Singh, Amit Sharma, George Hoppe, Weilin Song, Youstina Bolok, Jonathan E. Sears

**Affiliations:** 1 Cole Eye Institute, Cleveland Clinic, Cleveland, Ohio, United States; 2 Cellular and Molecular Medicine, Lerner Research Institute, Cleveland Clinic, Cleveland, Ohio, United States

**Keywords:** hypoxia inducible factor, retinopathy of prematurity, retinal metabolism, serine, 3-hydroxypyruvate

## Abstract

**Purpose:**

Transcriptional analysis of retina protected by hypoxia-inducible factor (HIF) stabilization demonstrates an increase in genes associated with aerobic glycolysis. We hypothesized that since protection is associated with a change in metabolism, oxygen-induced metabolites might transduce oxygen toxicity. We used global metabolic profiling to identify retinal metabolites increased in hyperoxia compared to normoxia.

**Methods:**

Untargeted gas chromatography mass spectroscopy (GC-MS) was performed on both mouse retina samples collected in hyperoxia and on primary human retinal endothelial cells, each with and without HIF stabilization. After identifying 3-hydropxypyruvate (3OH-pyruvate) as a unique hyperoxic metabolite, endothelial cells in culture and choroidal explants were challenged with 3OH-pyruvate in order to determine how this glycolytic intermediate was metabolized, and whether it had an effect on angiogenesis.

**Results:**

3OH-pyruvate was one of five metabolites at least 2.0-fold elevated in hyperoxia with a *P* value < 0.1. Once metabolized by endothelial cells, 3OH-pyruvate led to a 20-fold increase in 3-phosphoglycerate and a 4-fold increase in serine when cells were treated with Roxadustat to induce HIF stabilization. 3OH-pyruvate, but not pyruvate, destabilized HIF in endothelial cells with an increase in proline hydroxylation. 3OH-pyruvate was angiostatic in choroidal explant assays.

**Conclusions:**

3OH-pyruvate is a unique metabolite induced by hyperoxia that destabilizes HIF at least in part by a canonical pathway. 3OH-pyruvate induces angiostasis in vitro. HIF stabilization increases serine biosynthesis in vitro and in vivo.

Activating the transcription factor hypoxia-inducible factor (HIF) during the hyperoxic phase 1 of oxygen-induced retinopathy (OIR) prevents retinovascular growth attenuation and vascular obliteration in both mice and rats, as well as oxygen-induced lung disease in mice and premature baboons.^1^–^3^ This proangiogenic strategy using small molecule carbonyl glycines to pharmaceutically prevent catabolism of HIFα induces glycolysis by decreasing entry of pyruvate into the Krebs cycle through increased expression of pyruvate dehydrogenase kinase while increasing expression of the glucose transporter GLUT1 to facilitate glucose uptake.^4^ The conversion to aerobic glycolysis has been hypothesized to improve capillary survival because a glycolytic phenotype has been shown to be cytoprotective and angiogenic in endothelial cells. For example, endothelial tip cells proliferate when glycolysis is increased, as in the case of overexpression of phosphofructokinase B3.^5^

In this investigation, we tested the converse hypothesis: that hyperoxic metabolic intermediates themselves might initiate a signal that could retard growth of retinal blood vessels during hyperoxia, specifically by their interaction with HIF signaling. In order to understand how metabolic plasticity relates to oxygen-induced vascular injury and conversely how HIF stabilization induces protection, we used global metabolic profiling to identify retinal metabolites increased in hyperoxia compared to normoxia. One of these metabolites, 3OH-pyruvate, is a substrate of GRHPR, a glycolytic enzyme upregulated by HIF stabilization in the retina.^6^ This investigation identified 3OH-pyruvate to be a hyperoxia-only metabolite and evaluated the effect of 3OH-pyruvate on HIF stability, angiogenesis, and endothelial cell metabolism.

## Methods

### OIR, Endothelial Cell, and Angiogenesis Models

Animals used in our experiments were treated under a protocol approved by the Cleveland Clinic Institutional Animal Care and Use Committee (IACUC, protocol # 2016-1677) and adhered to the ARVO Statement for the Use of Animals in Ophthalmic and Vision Research. The OIR model by Smith causes retinovascular growth attenuation and vascular obliteration (phase 1 hyperoxic ischemia) leading to neovascularization (phase 2 hypoxic ischemia).^7^ Retinal endothelial cells were used in primary culture in order to perform untargeted GC-MS studies. The choroidal explant assay using postnatal day (P)8 mice was used to determine the effect of 3OH-pyruvate on angiogenesis.

### Reagents

LC-MS grade methanol (HiPerSolv CHROMANORM; BDH VWR International, Radnor, PA, USA) and LC-MS grade chloroform (LiChrosolv; MilliporeSigma, Burlington, MA, USA) were used for all the metabolite extractions. Methoxyamine hydrochloride, potassium salt of 3OH-pyruvate, pyruvate, anhydrous pyridine, n-alkanes, and all the analytical standards used to prepare the metabolite library were purchased from Sigma-Aldrich Corp. (St. Louis, MO, USA). Roxadustat (FG-4592) was purchased from AdooQ BioSci (Irvine, CA, USA). MSTFA was purchased from Macherey-Nagel (Bethlehem, PA, USA). Ultrapure water (MiliQ; MilliporeSigma) with resistivity 18.2 MΩ cm was used in all the experiments. Hanks' balanced salt solution (HBSS) without calcium chloride, magnesium chloride and phenol red, PBS, and Dulbecco's modified Eagle's medium (DMEM) were from the Cleveland Clinic Media Lab (Cleveland, OH, USA).

### OIR Mouse Model

The OIR model of ROP was based on a previously described method.^7^ Mice were placed in 75% hyperoxia with their nursing dam from P7 to P12 and removed to room air, unless experiments were terminated at P10. The HIF prolyl hydroxylase inhibitor Roxadustat was administered three times intraperitoneally (IP) on P6, P8, and P10.^8^ Retinas were harvested on P10 after 6 hours of the third Roxadustat injection. Control mice were administered the drug diluent (i.e., PBS).

### Metabolite Extraction From Retina

As described above, animals used in our experiments were used in procedures approved by the Cleveland Clinic IACUC (protocol # 2016-1677). C57BL/6J wild-type mice were provided the Jackson Laboratory (Bar Harbor, ME, USA). Animals were euthanized by an overdose of IP ketamine/xylazine and retinas were isolated using the quick retina harvesting method described earlier by Winkler.^9^ One eye at a time was proptosed out of the orbit using forceps and held from the optic nerve. Using a sharp single-edge blade, the eye was incised along the equator and the retina allowed to quickly exit the posterior cavity. Retinas were washed with cold HBSS and were snap-frozen in liquid nitrogen, and then stored at −80°C until further use.

Retinas were thawed on ice then homogenized in a 1.5-mL tube with a disposable fitted pestle in 50 μL of −20°C cold 50% methanol. An additional 750 μL of −20°C cold 50% methanol was added to the lysate. We then added 400 μL of −20°C chloroform to the samples, vortexed briefly, and shaken at 1400 rpm for 30 minutes at 48°C (Thermomixer C; Eppendorf, Hamburg, Germany), followed by centrifugation at 15,000*g* at 4°C for 5 minutes to separate the different phases. We dried and stored 300 μL of upper phase containing polar metabolites at −80°C until further use.

### Sample Derivatization for GC-MS

Samples stored at −80°C were again dried briefly for 10 minutes at 25°C under vacuum, dissolved in 25 μL solution of 40 mg mL^−1^ methoxyamine hydrochloride in pyridine and incubated at 45°C for 30 minutes with vortexing (Thermomixer C, Eppendorf) at 1000 rpm. Samples were then allowed to cool at room temperature, followed by addition of 25 μL MSTFA (Macherey-Nagel, Düren, Germany) and incubation at 45°C for 30 minutes with vortexing (Eppendorf) at 1000 rpm.

### Untargeted GC-MS Method

GC-MS analysis was performed on a GC device (6890N GC; Agilent, Santa Clara, CA, USA) coupled to mass selective detector (MSD; Agilent) equipped with an electron impact ionization source set at 70 eV. The source was held at a constant temperature of 230°C and quadrupole set at constant temperature of 150°C. We injected 1 μL of sample into GC installed with DB-17MS (30 m × 0.25 mm × 0.25 μm) column with a constant flow of helium as carrier gas at 1.5 mL min^−1^; injector temperature set to 270°C. GC parameters used for the separation of metabolites were: initial temperature 60°C for 1 minutes followed by ramping of temperature with a ramp rate of 6°C min^−1^ to a final temperature of 320°C held for 10 minutes. MSD transfer line temperature was set to 280°C. Full scans were acquired with quadrupole mass analyzer set to scan m/z 50-700. Each batch of samples was injected along with a blank after every 2 to 4 samples. For retention index determination, underivatized n-alkanes mix (C10-C40, even numbered, 50 mg L^−1^ each; Sigma-Aldrich Corp.) was injected in the beginning and at the end of each day's run.

### Data Analysis

**Figure 1:**
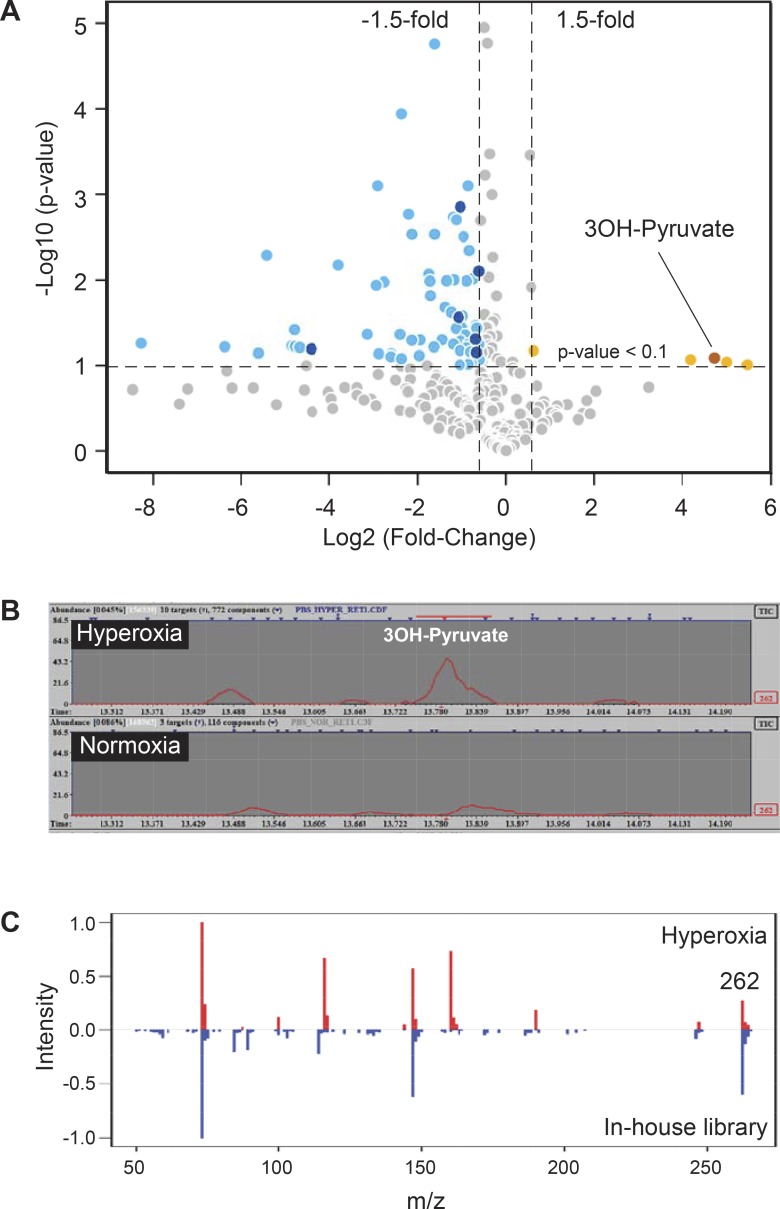
Hyperoxia-induced accumulation of 3OH-pyruvate in the retina. (A) Volcano plot representing changes in retinal content of metabolites isolated from mouse pups subjected to 75% oxygen (hyperoxic, n = 6; normoxic, n = 4). Identified compounds that are decreased and increased are represented by darker blue and brown circles, respectively and listed in the Table. (B) Extracted ion chromatogram for m/z 262 depicting increased concentration of 3OH-pyruvate in hyperoxic retina. (C) Head-to-tail spectral comparison of 3OH-pyruvate in retina to an in-house metabolite library.

Data were analyzed with metabolite detector software^10^ and metabolite identities were determined by matching retention index and spectra of each peak in the sample against in-house and Golm libraries.^11^ Software used for statistical analysis and to prepare the volcano plot analysis included metaboanalyst^12^ and R version 3.4.0 2017–04–21.^13^ AMDIS software was used for pictorial representation of the chromatogram in Figure 1B.^14^ Chemical drawing software (ACD ChemSketch, version 2017.1.2; Advanced Chemistry Development, Inc., Toronto, ON, Canada) was used to prepare the chemical structures for Figure 6A.

### Quantification of 3OH-Pyruvate

Once 3OH-pyruvate was established as the only hyperoxia metabolite identifiable by GC-MS, its concentration in vivo was estimated from pooled samples obtained from eight retinas. Metabolites were extracted from the retina, as described earlier, with methanol/chloroform/water extraction. Isotope dilution mass spectrometry method was used for quantification.^15^ Pooled retina samples and calibration standards were all spiked with equal quantities of U-^13^C Ribitol as an internal standard. A peak height of m/z 262 corresponding to 3OH-pyruvate in sample and standards were normalized to one of the high abundance U-^13^C Ribitol fragments with m/z 323.

### Cell Culture

Human microvascular retinal endothelial cells (REC) were procured from Cell Systems (Kirkland, WA, USA) and maintained in medium (Complete Classic Medium with serum and CultureBoost; Cell Systems). Spontaneously immortalized human Müller cell line MIO-M1 was a gift (G. Astrid Limb; UCL Institute of Ophthalmology, London, UK) and was maintained in DMEM (CC Media Lab) supplemented with 10% fetal bovine serum (Gibco Thermo Fisher Scientific). Cells were cultured in 37°C incubator with 5% CO_2_.

### Gene Expression Assay by Quantitative PCR

REC or MIO-M1 cells were grown in 60-mm Petri dishes to subconfluency, treated with 1 mM glyoxylate or 3OH-pyruvate overnight, harvested in RLT lysis buffer (Qiagen, Germantown, MD, USA) supplemented with 1% β-mercaptoethanol (Pharmacia Biotech, Uppsala, Sweden) and homogenized by passing through QIAshredder (Qiagen). Total RNA was isolated using an RNA extraction kit (RNeasy Mini kit; Qiagen) and reverse transcription was performed using a synthesis kit (Verso cDNA Synthesis Kit; Thermo Fisher Scientific, Waltham, MA, USA) according to manufacturer's instructions. Relative mRNA content was measured by quantitative PCR using Radiant SYBR Green Hi-ROX Kit (Alkali Sci, Fort Lauderdale, FL, USA) and primer sets (QuantiTect; Qiagen) validated for the gene of interest *PSAT1* or internal control *HPRT1* according to the manufacturer suggested protocol. Data was expressed as *PSAT1* mRNA content normalized to *HPRT1* and relative to control levels (without treatment).

### Metabolite Extraction From Cultured Cells

REC cells were grown to subconfluency in six-well plates and treated with 3OH-pyruvate, pyruvate, or PBS for 3 hours. To study the effect the Roxadustat, cells were pretreated with 10-μg mL^−1^ Roxadustat for 1 hour and prior to 3 hours incubation with 5 mM 3OH-pyruvate or 5 mM pyruvate or nothing. Intracellular metabolites were extracted as described previously.^15^ Briefly, cells were washed with normal saline to remove the traces of media. To the washed cells, 400 μL of −20°C cold methanol and 400 μL of cold water were added, and cells were scraped with a 1.8-cm blade cell scraper (Costar-Corning, Inc., Corning, NY, USA). Cell lysates were transferred to 1.5-mL tubes prefilled with −20°C cold chloroform and vortexed (Eppendorf) at 1400 rpm for 30 minutes at 4°C. Samples were then centrifuged at 15,000*g* for 5 minutes at 4°C. A total of 300 μL of upper phase containing polar metabolites was dried under vacuum at −4°C and stored at −80°C until further use.

### Western Blotting

REC or MIO-M1 cells were plated in 60 mm Petri dishes and grown to subconfluency. On the day of the experiment fresh media was provided containing treatments of Roxadustat (AdooQ BioSci, Irvine, CA, USA), 3OH-pyruvate, pyruvate, glyoxylate (all from Sigma-Aldrich Corp.) or MG-132 (MilliporeSigma) as described in figure legends. Cell proteins were extracted by lysing cell monolayers with RIPA buffer (Sigma-Aldrich Corp.) containing protease inhibitors cocktail Complete (Roche Diagnostics, Mannheim, Germany) followed by centrifugation at 20,000*g*, for 15 minutes at 4°C and collecting supernatants. Cellular proteins were denatured by heating 3 minutes in 2X sample buffer (Novex SDS; Invitrogen, Carlsbad, CA, USA) and resolved on gradient 4% to 20% polyacrylamide precast gels (Invitrogen) and in running buffer (Tris-Glycine SDS; Bio-Rad Laboratories, Hercules, CA, USA) along with prestained molecular weight markers (SeeBlue Plus2; Invitrogen). Proteins then were electrotransferred overnight at 30 V in Tris-Glycine transfer buffer (Bio-Rad Laboratories) to polyvinylidene fluoride (PVDF) membranes (FluoroTrans W; Pall Corp, Port Washington, NY, USA), blocked with 5% nonfat dry milk (Bio-Rad Laboratories) and incubated overnight at 4°C with the following primary antibodies: HIF-1α (cat #10006421; Cayman Chemical Company, Ann Arbor, MI, USA) or hydroxylated HIF-1α (cat # 3434, Cell Signaling Technologies, Danvers, MA, USA). Protein bands were revealed by immunochemiluminescene using corresponding secondary antibodies conjugated with horseradish peroxidase (Jackson ImmunoResearch Labs, West Grove, PA, USA), enhanced chemiluminescence (ECL) substrate (Western Lightning Plus-ECL; PerkinElmer, Waltham, MA, USA) and x-ray film (CL-XPosure; Pierce Biotechnology, Rockford, IL, USA).

For quantitative analysis of HIF bands, same protein samples were subjected to the identical SDS-PAGE protein separation as above, but immunoblotting procedure with fluorescence detection was performed using reagents and methods obtained from LI-COR Biotechnology (Lincoln, NE, USA). Specifically, PVDF membranes were blocked with blocking buffer (Odyssey; LI-COR Biotechnology), probed with anti-β-actin goat antibody (cat # sc-1615; Santa Cruz Biotechnology, Inc., Dallas, TX, USA) in addition to the one of the above-mentioned HIF-1α or hydroxylated HIF-1α rabbit antibodies and detected by corresponding secondary antibodies conjugated with near-infra red fluorescent dyes IRDye 680RD (anti-goat) and IRDye 800CW (anti-rabbit). Densitometry was performed in imaging software (Image Studio, version 5.2.5; LI-COR Biotechnology) and data expressed as the ratio hydroxylated/total HIF-1α normalized to β-actin.

### Choroidal Sprout Outgrowth Assay

Ex vivo choroidal tissue cultures were established essentially as described by Shao et al.^16^ and endothelial cell sprouting was quantified with ImageJ (http://imagej.nih.gov/ij/; provided in the public domain by the National Institutes of Health, Bethesda, MD, USA) as the area of sprout outgrowth. Specifically, eyes of 8-day-old C57BL/6J mouse pups were used to dissect apart the RPE-choroid-sclera complex, which was cut into 1 × 1 mm pieces and immobilized on the bottom of six-well tissue culture clusters with grow factor reduced Matrigel (cat # 356231, Corning, Inc.). All procedures involving live mice were approved by the Cleveland Clinic IACUC (protocol # 2016-1677). Choroidal explants were cultured in 1.5 mL of medium (Cell Systems) supplemented with penicillin and streptomycin. Two days after plating, cultures were treated with pyruvate or 3OH-pyruvate as detailed in the figure legend for another 2 days. Live microscopy images were taken at the start (day 2) and the end (day 4) of the treatments. Using thresholding tool in ImageJ, the area of sprouts outgrowth was isolated, measured in pixel number, and expressed as fold-change from day 2 to day 4.

## Results

### 3OH-Pyruvate Is a Retinal Metabolite Increased in Hyperoxia

In order to determine whether hyperoxia induced specific retinal metabolites, global metabolic profiling was obtained by placing P7 mouse pups with their nursing dam into 75% oxygen for 3 days and their retinas quickly dissected and metabolites extracted. A volcano plot (Fig. 1A) derived from the untargeted GC-MS analysis of metabolites with fold changes greater than 2 and *P* value < 0.1 calculated using *t*-test revealed four compounds that were increased in response to hyperoxia. Using an in-house library comprised of 40 standards, we confirmed the identity of one of these compounds to be 3OH-pyruvate (Figs. 1B, 1C). Untargeted GC-MS revealed a peak at m/z 262 which corresponded exactly to an in-house library containing 3OH-pyruvate with a cumulative score of 0.75, calculated using retention index and spectral match (Figs. 1B, 1C). The molecular weight of 1MeOX 2TMS derivative of 3OH-pyruvate is 277; the standard loss of one methyl group upon electron impact ionization created the main fragment with m/z 262. The concentration of 3OH-pyruvate in the retina in vivo was in the range of 10 pM, which was above the limit of detection, but lower than the estimated limit of quantifications. 3OH-pyruvate was seen exclusively in hyperoxic retina (Fig. 1B).

### 3OH-Pyruvate Increases Phosphoserine Aminotransferase Expression (PSAT1)

**Figure 2:**
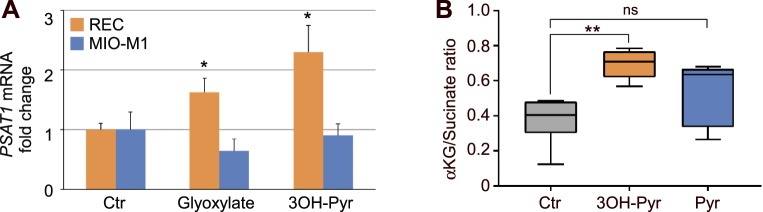
(A) Cell-specific induction of PSAT1 mRNA by 3OH-pyruvate. REC or MIO-M1 cells were treated overnight with 1 mM 3OH-pyruvate or glyoxylate, total RNA was isolated and analyzed for PSAT1 mRNA normalized to HPRT1 mRNA by RT-qPCR and expressed as fold-change versus untreated controls (n = 5 for each condition). (B) Increased αKG/succinate ratio in 3OH-pyruvate treated REC cells measured by GC-MS (see Methods for the details). Ctr, control; 3OH-Pyr, 3OH-pyruvate; Pyr, pyruvate (n = 6 for each condition). *P < 1 × 10^−5^; **P < 5 × 10^−6^ versus control.

Hyperoxic retina also showed decreases in multiple amino acids including serine (double silylated, 2 TMS, Table), which can be synthesized from 3OH-pyruvate. The decrease in hyperoxic retinal serine suggested that hyperoxia-induced downregulation of HIF also downregulated key enzymes within the serine synthesis pathway that might be regulated by HIF. We previously determined that retinal glyoxylate reductase/hydroxypyruvate reductase (GRHPR) and phosphoglycerate mutase (PGAM), two enzymes that participate in serine synthesis are induced by HIF.^6^ Conversely, PSAT1, an enzyme that transfers an amino group to 3OH-pyruvate from glutamate to form serine, was not upregulated in our previous experiments of in vivo HIF stabilization.^6^ We reasoned that if an increase in *PSAT1* expression was limited to endothelial cells, RNAseq of whole retina might not detect it, as endothelial cells are a small fraction of total cell volume as over half the retina is avascular. Therefore, to determine if *PSAT1* expression and catabolism of 3OH-pyruvate was specific to endothelial cells, we treated REC cells with 3OH-pyruvate and quantified *PSAT1* expression in comparison to Müller cells. *PSAT1* mRNA was increased 2-fold in RECs alone by both glyoxylate (another substrate of GRHPR) and 3OH-pyruvate (Fig. 2A). On the contrary, Müller cells demonstrated no increase in *PSAT1* mRNA (Fig. 2A).

**Table:** Retinal Metabolites Affected by Hyperoxia

Metabolite	Fold-Change From Normoxia	*P* Value
3OH-pyruvic acid (1MEOX) (2TMS)	26.78	0.082
Malic acid (3TMS)	0.658	0.008
Valine (1TMS)	0.634	0.070
Serine (2TMS)	0.624	0.049
Oxalic acid (2TMS)	0.489	0.001
Isoleucine (1TMS)	0.476	0.027
Lysine (4TMS)	0.047	0.064

Metabolites identified using in-house metabolite library, with a fold-change (hyperoxic retina/normoxic retina) greater than or less than 1.5-fold and with *P* value less than 0.1, calculated using unequal variance *t*-test. Only positively identified metabolites are listed.

### 3OH-Pyruvate Treatment Increases α-Ketoglutarate

Alpha-ketoglutarate (αKG), a product of glutamate transamination and a necessary cofactor for HIF prolyl-hydroxylase (HIF-PHD) induced downregulation of HIFα, was measured as part of the αKG to succinate ratio and was increased in RECs exposed to 3OH-pyruvate (Fig. 2B).

**Figure 3:**
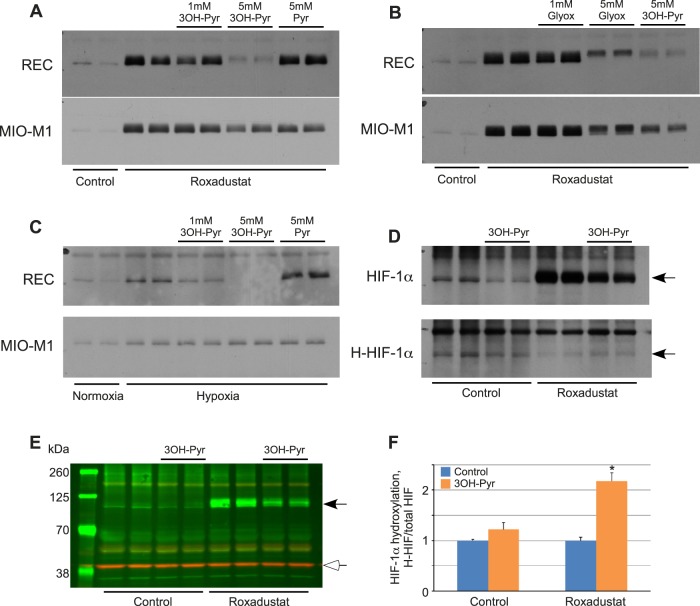
Effect of 3OH-pyruvate on the cellular levels of HIF-1α and its hydroxylation. (A–C) REC or MIO-M1 cells were first treated with 10 μg mL^−1^ Roxadustat for 2 hours (A, B) or 2% O_2_ (hypoxia, [C]) followed by addition of indicated amounts of 3OH-pyruvate, glyoxylate, or pyruvate without changing media (n = 6 for each condition). Cellular proteins were extracted and probed for HIF-1α protein levels by Western blot. (D) REC cells were treated with or without 10 μg mL^−1^ Roxadustat, 5 mM 3OH-pyruvate in the presence of 10 μM MG-132 for 4 hours (n = 6 for each condition). Cellular proteins were extracted and probed for HIF-1α or hydroxylated HIF-1α (H-HIF) protein levels by Western blot. (E) Representative Western blot image of simultaneous detection of HIF-1α (black arrowhead) and β-actin (white arrowhead). (F) Ratio of hydroxylated/total HIF-1α normalized to β-actin and expressed as fold-change in 3OH-Pyr treated versus untreated REC cells ± SEM obtained by densitometry as described in Methods (n = 4 for each condition). *P < 0.001 versus control.

The REC specific increase in α-KG/succinate ratio suggested that 3OH-pyruvate might affect the stability of the HIFα subunit in RECs, but not in Müller cells. We tested this hypothesis using retinal endothelial cells and Müller cells exposed to hypoxia mimesis by Roxadustat to demonstrate the dose dependent destabilization of HIF-1α under 3OH-pyruvate exposure (Fig. 3A) or glyoxylate (Fig. 3B). HIF downregulation was specific to retinal endothelial cells but not Müller cells and was not seen using either PBS or pyruvate as control stimuli. Figure 3C analogously shows the destabilizing effect of 3OH-pyruvate on HIF stability but demonstrates this effect in hypoxia rather than under pharmaceutical HIF stabilization. The destabilization of HIF likely followed at least in part the canonical pathway utilizing HIF-PHDs and the αKG cofactor as we were able to capture enhanced proline hydroxylation of HIF-1α using an inhibitor of the proteasome (MG132) and an antibody specific to hydroxylated proline of HIF-1α (Fig. 3D). An actin control (Fig. 3E) confirms that equal protein is loaded in each well. Quantification of hydroxylated HIF proline is shown in Figure 3F.

### 3OH-Pyruvate Prevents Angiogenesis

**Figure 4:**
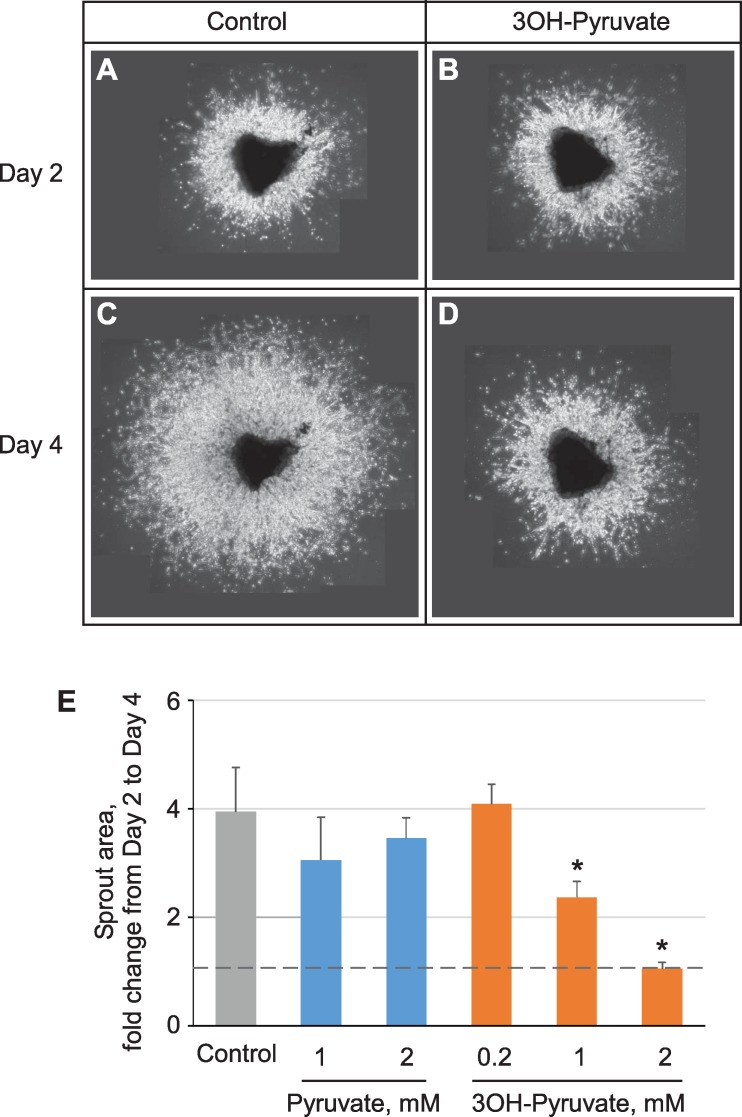
Angiostatic effect of 3OH-pyruvate on choroidal sprouting. (A–D) Representative images of 2- (A, B) and 4-day-old (C, D) choroidal endothelial outgrowth (treated 2 days after plating) either untreated (A, C) or subjected to 2 mM 3OH-pyruvate. (E) Quantification of the sprout area (see Methods for details) following 2 days of growth in the presence of indicated amounts of pyruvate or 3OH-pyruvate. Data presented as the area fold-change from day 2 (dashed line) to day 4 (Control n = 5, 1 mM 3OH-Pyruvate n = 6, all other n = 3). *P < 0.001 versus control.

We next treated choroidal explants with 3OH-pyruvate to see if there was a direct effect on angiogenesis, given the finding that it inhibits HIF stabilization. Choroidal explants were taken from P8 mice and established on a Matrigel matrix and exposed to 3OH-pyruvate or pyruvate or PBS. One mM 3OH-pyruvate induced partial and 2 mM 3OH-pyruvate induced complete angiostasis (blocked angiogenesis) while neither dose of pyruvate was angiostatic (Fig. 4).

### HIF Stabilization Promotes Conversion of 3OH-Pyruvate to Serine

**Figure 5:**
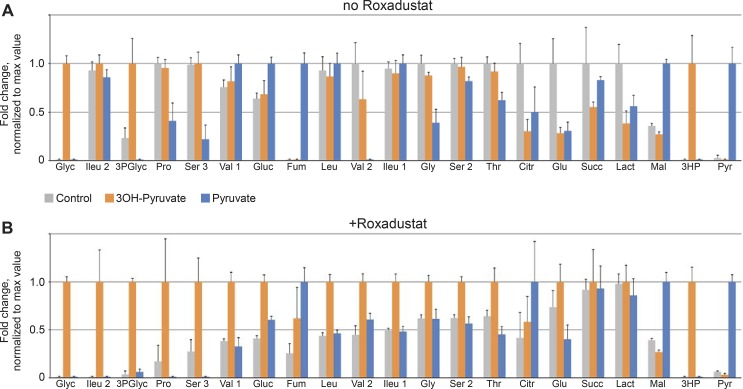
Changes of selected metabolites levels in REC cells induced by either 3OH-pyruvate or pyruvate in the (A) absence (n = 6 for all the samples) or (B) presence (n = 6 for all the samples) of Roxadustat and expressed as fold-change normalized to maximal value for each individual metabolite (see Methods for the details). 3HP, 3-hydroxy pyruvic acid (1MEOX) (2TMS); 3PGlyc, glyceric acid-3-phosphate (4TMS); Citr, citric acid (4TMS); Fum, fumaric acid (2TMS); Glu, glutamic acid (3TMS); Gluc, glucose (1MEOX) (5TMS); Gly, glycine (3TMS); Glyc, glyceric acid (3TMS); Ileu 1, isoleucine (1TMS); Ileu 2, isoleucine (2TMS); Lact, lactic acid (2TMS); Leu, leucine (1TMS); Mal, malic acid (3TMS); Pro, proline (1TMS); Pyr, pyruvic acid (1MEOX) (1TMS); Ser 2, serine (2TMS); Ser 3, serine (3TMS); Succ, succinic acid (2TMS); Thr, threonine (3TMS); Val 1, valine (1TMS); Val 2, valine (2TMS).

The effect of HIF stabilization on the levels of multiple metabolites was next tested in RECs given either 3OH-pyruvate or pyruvate. 3OH-pyruvate but not pyruvate dramatically increased glycerate and 3-phosphoglycerate with and without HIF stabilization, whereas HIF stabilization in the presence of 3OH-pyruvate promoted a 2-fold accumulation of serine, as well as other amino acids such as isoleucine, leucine, valine, and glycine (Fig. 5). Pyruvate treatment did increase 2-fold the concentration of malate and fumarate; the latter especially in the absence of HIF stabilization, suggests that pyruvate alone without HIF activity fueled oxidative phosphorylation via the TCA cycle. 3OH-pyruvate doubled glucose levels under HIF stabilization.

**Figure 6:**
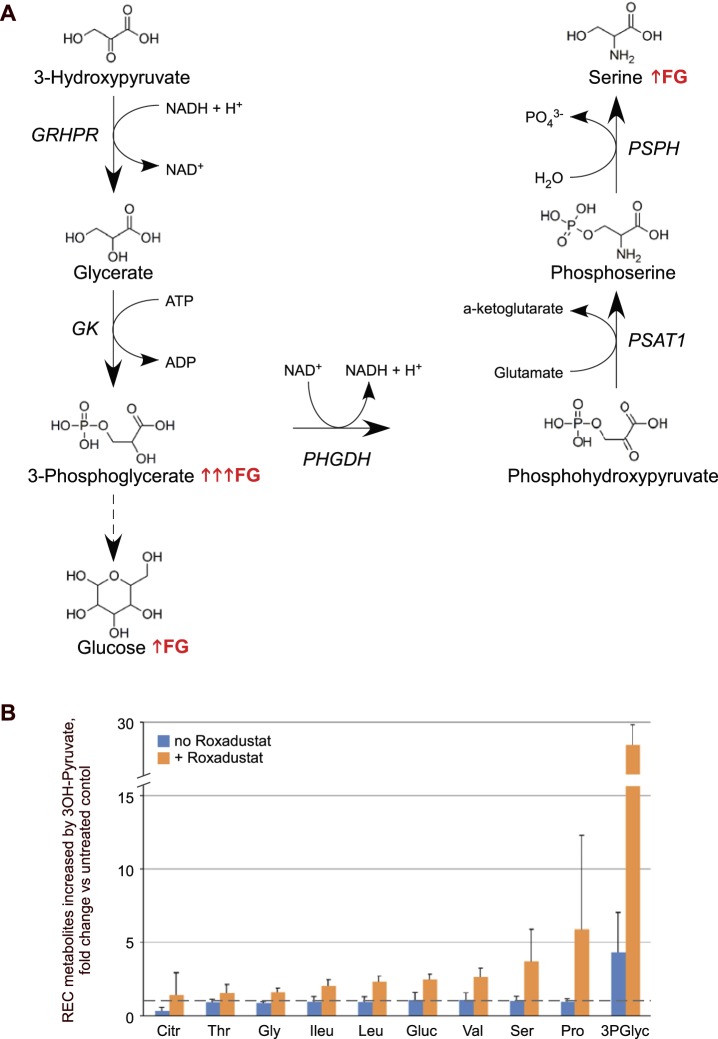
Breakdown of 3OH-pyruvate in REC cells leads to Roxadustat-induced serine synthesis and gluconeogenesis. (A) Schematic representation of metabolic pathways involving catabolism and utilization of 3OH-pyruvate in REC cells that are particularly enhanced in the presence of Roxadustat (↑FG or ↑↑↑FG in case of 3-phosphoglycerate). (B) Roxadustat-induced changes of select metabolites in 3OH-pyruvate–treated REC cells expressed as fold-change versus untreated control (dashed line). 3PGlyc, glyceric acid-3-phosphate; Citr, citric acid; Gluc, glucose; Gly, glycine; Ileu, isoleucine; Leu, leucine; Pro, proline; Ser, serine; Thr, threonine; Val, valine.

A summary of the effect of these increased metabolites on the serine biosynthesis pathway is presented in Figure 6A, alongside Roxadustat-induced changes of select metabolites in 3OH-pyruvate-treated REC cells expressed as fold-change versus untreated control (dashed line, Fig. 6B). Note once more that HIF stabilization increases serine synthesis.

## Discussion

We report a novel link between metabolic plasticity and HIF-dependent regulation of angiogenesis. Our investigation does not conclusively prove that 3OH-pyruvate is a dominant mediator of oxygen toxicity; there are likely multiple parallel pathways that mediate toxicity. This finding does however make plausible that 3OH-pyruvate plays a larger role than simply a precursor to D-glycerate. 3OH-pyruvate is reported to inhibit pyruvate decarboxylase and is a putative factor associated with insulin resistance in Type 2 diabetes.^17^,^18^

We do not know the source of 3OH-pyruvate in hyperoxic retina. 3OH-pyruvate can be produced by D-amino acid oxidase or by serine-pyruvate transaminase.^19^,^20^ In plants, 3OH-pyruvate is an intermediate of the dark cycle, which relies on Rubisco to fixate carbon dioxide.^21^ 3OH-pyruvate is a substrate of GRHPR, which converts either glyoxalate to glycolate or 3OH-pyruvate to D-glycerate. Deletion of GRHPR underlies the gene defect in patients with primary hyperoxalurias Type II.^22^

We also note that there was not stoichiometric conversion of 3OH-pyruvate to serine and that the levels of 3OH-pyruvate in retina were small. We speculate that a large fraction of D-glycerate and 3-phosphoglycerate might be used for gluconeogenesis or converted to phosphoenolpyruvate for entry into the glycolytic pathway. We also observe that a relatively high concentration of 3OH-pyruvate is necessary to induce HIF destabilization in cell culture. We rationalize that unlike pyruvate, 3OH-pyruvate is not easily transported across cell membranes but within the cytosol its biological effect is potent. Other investigations use similar 3OH-pyruvate concentrations. Williamson and Ellington demonstrate that the rat liver when supplemented with up to 2 mM 3OH-pyruvate can utilize it as a substrate to produce glucose via the gluconeogenic pathway; 5 mM 3OH-pyruvate was needed to inhibit pyruvate decarboxylase mentioned in the preceding paragraph.^23^

Our data demonstrates that 3OH-pyruvate increases αKG, which is a cofactor to the oxygen sensitive HIF-PHD. The post-translational catabolism of the HIFα subunit is initiated by HIF-PHD, which catalyzes a trans-4 prolyl hydroxylation at Pro402 and Pro564 within the oxygen-dependent degradation domain of HIF-1α and HIF-2α.^24^–^26^ Once hydroxylated, the HIFα subunit becomes a substrate for the Von Hippel-Lindau E3 ubiquitin ligase. Polyubiquination of HIFα targets the subunit to the proteosome, providing a rapid post-translational downregulation of HIF activity. An increase in αKG might explain the destabilization of HIFα by 3OH-pyruvate. Dioxygenases such as HIF-PHDs catalyze the splitting of molecular dioxygen to hydroxylate proline and αKG to form succinate and carbon dioxide.^27^ There are multiple reports of metabolites regulating HIF activity in addition to our finding that 3OH-pyruvate modulates HIF stability. Fumarate and succinate increase expression of HIF dependent gene products by inhibiting TET proteins, which are another class of αKG dependent enzymes.^28^ And in tumors with succinate dehydrogenase deficiency, succinate accumulation is considered a HIF stabilizing environment because it is a product of trans-4-prolyl-hydroxyation of HIF and should therefore inhibit the forward reaction associated with enzyme activity.^29^,^30^

Hyperoxia *downregulates* and HIF stabilization *upregulates* serine and glycine production. Serine may play a critical role in retinovascular homeostasis as mutations in PHGDH and low serum serine levels are associated with macular telangiectasia Type 2.^31^ Serine is demethylated to glycine in the cytosol or mitochondria in the one-carbon cycle to produce formate.^32^ The additional reducing equivalent is available to protect glutathione from oxidation or adduction to susceptible thiol residues forming protein-S-glutathione disulfide bonds that change protein conformation.^33^ Our previous studies and our data here demonstrate that enzymes important for 3OH-pyruvate conversion to serine (GRHPR, PHGDH, and PSAT) are either increased under HIF stabilization or by excess 3OH-pyruvate. On the other hand, the decrease in serine during hyperoxia may implicate declining fluxes from cytosolic and mitochondrial folate pathways under excess oxygen. Accumulation of 3OH-pyruvate therefore may reflect a decrease in serine synthesis during hyperoxia.
